# Missouri

**Published:** 1896-04

**Authors:** 


*Missouri.* St. Louis is agitating the passage of a law creating a rigid inspectorship of milk and dairies. It calls for all dairies to be removed outside of the city limits.

